# A Simple Method of Transfusion

**Published:** 1897-03

**Authors:** Hunter P. Cooper

**Affiliations:** Atlanta, Ga., Professor of Anatomy and Clinical Surgery, Atlanta Medical College


ATLANTA

Medical and Surgical Journal.

VOL. XIV. MARCH, 1897. No. 1.

LUTHER B. GRANDY, M.D., MANAGING EDITOR.

M. B. HUTCHINS, M.D., BUSINESS MANAGER.

COLLABORATORS:
A. W. CALHOUN, M.D., LL.D., VIRGIL O. HARDON, M.D., FLOYD W. McRAE, M.D., JOSEPH EVE ALLEN, M.D., AUGUSTA, GA., AND HENRY R. SLACK, PH.M., M.D., LAGRANGE, GA.

### ORIGINAL COMMUNICATIONS.

### A SIMPLE METHOD OF TRANSFUSION.

BY HUNTER P. COOPER, M.D., ATLANTA, GA.,
Professor of Anatomy and Clinical Surgery, Atlanta Medical College.

Probably all who have made intravenous injections of a saline solution have been confronted by this difficulty—when there has been severe hemorrhage or profound shock, the median basilic vein is found empty and collapsed. In many cases of severe hemorrhage it is difficult to find the vein after making the incision over it, and much greater is the difficulty of inserting the needle into the vein in order to make the injection. Recently I attempted to inject a saline solution into the median basilic vein of a patient who was suffering profoundly from shock and loss of blood following resection of the rectum. As usual, I tied a bandage around the upper part of the arm in order to obstruct the venous current and cause the median basilic vein to come into view; it was impossible to see it at all. I then made an incision over the situation of the vein and finally exposed it thoroughly. It was apparently completely empty and collapsed. I attempted to puncture it with my needle in order to make the injection, but the point of the needle passed into the posterior wall of the vein and I had to withdraw it and try at a little higher point; at this effort the needle penetrated entirely through the vein. I then gave up any further attempt and resorted to subcutaneous injections over different parts of the body. I had this same difficulty a number of times on account of the collapsed condition of the vein. To meet this condition I have devised a special needle and the following technique:

The needle is simply a probe-pointed needle with an opening in the middle of the end, and a constriction above the end; in every other respect like an ordinary aspirating needle. Having exposed the vein and dissected it up from the tissues underneath it for a full inch, apply pressure forceps to it an inch apart; then cut through the vein at a point near the distal forceps, insert the probe-pointed needle into the proximal cut end of the vein, and secure it in the vein by a silk ligature; tie tightly around the needle at the constricted portion of the same, then remove the compression forceps and make the injection. After the injection ligate both of the cut ends with catgut and close up the wound.

This method possesses advantages over the old method for the reason that it is always a perfectly simple matter to get into the vein whether it is full or empty. It has advantages over the use of a saline fluid subcutaneously as, in the first place, it is painless, and in the second place, the fluid goes directly to the heart without any absorption having to take place as in the subcutaneous method.

There are several ways of increasing the bulk of blood after a hemorrhage. In the first place, nature's plan is the one that we try to imitate. After a hemorrhage the vessels soak up all the fluid in the cellular spaces, and in severe hemorrhage with shock you need to aid nature, and this is done by the transfusion of saline injections. In order to get a saline fluid into the blood several methods can be employed. One is to inject the solution direct into the vein, and by this you get almost immediate results, as it does not require any absorptive power. It goes direct from the veins into the right auricle of the heart, then into the right ventricle, and after passing through the pulmonary circulation is forced by the heart into the general circulation. If the heart is beating at all, it enters almost immediately into the arteries, and by supplying the brain prevents collapse.

Another way is by the rectal injection of a saline solution. This is a very good method and does not interfere with the method just mentioned, as the nurse or attendant can give this injection while you are preparing the other. The injection of about a quart into the rectum after a severe operation is one of the most useful procedures. However, one objection to it is that it is a very slow process in its results, as the absorption of it depends upon the activity of the heart, and when that is very feeble it is absorbed very slowly. It is a slow method and one upon which you cannot count when in a hurry.

Another method is the subcutaneous injection of large amounts of a saline solution over the thigh, abdomen, chest, etc. In this way you can inject from a quart to three pints, and this is absorbed more quickly than when put into the rectum, for in the rectum it has to pass through the mucous membrane, while here it is in the intercellular spaces and the absorption is therefore greater.

These are the three methods: The intravenous injection, the rectal, and the hypodermic. The beauty of the rectal is that it can be given in connection with the other methods. Of all the different methods I prefer the intravenous, as the fluid goes directly into the circulation. I also prefer it for the reason that it is quick. The technique is so simple that in five minutes the solution is flowing into the vein.

The needle was made by Stohlmann, Pfarre & Co., of New York City, and was made to go with Allen's surgical pump. I keep the fluid at 110° by immersing the container in water and watching the thermometer. It takes about twenty minutes to throw one pint into the circulation with this needle."

---

THE best way to remove blood-stains is to soak the towels, etc., in warm water to which a teaspoonful of tartaric acid has been added. No soap is needed.—*Ex.*

